# *Thismia sitimeriamiae* (Thismiaceae), an extraordinary new species from Terengganu, Peninsular Malaysia

**DOI:** 10.3897/phytokeys.179.68300

**Published:** 2021-06-29

**Authors:** Mat Yunoh Siti-Munirah, Nikong Dome, Chris J. Thorogood

**Affiliations:** 1 Forest Research Institute Malaysia, 52109, Kepong, Selangor, Malaysia; 2 DigitalDome Photography, 21500 Permaisuri, Terengganu, Malaysia; 3 Department of Plant Sciences, University of Oxford, South Parks Road, Oxford, UK; 4 University of Oxford Botanic Garden, Rose Lane, Oxford, UK

**Keywords:** conservation, forestry, mycoheterotrophy, species diversity, taxonomy

## Abstract

We describe an extraordinary new species in the genus *Thismia* from the Terengganu State of Malaysia in the Malay Peninsula. The new species, which we name *Thismia sitimeriamiae*, is distinct from all other *Thismia* species known to science, most notably in its unique mitre configuration formed by the inner tepals and its floral surface morphology characterised by conspicuous orange trichomes. We discuss our findings in the context of underestimated species diversity in the genus *Thismia* and implications for their conservation. We recommend assigning *T. sitimeriamiae* the conservation status as Critically Endangered (CR) according to IUCN criteria.

## Introduction

The Thismiaceae is a highly unusual family of flowering plants belonging to the order Dioscoreales (APG IV 2016). There are currently 86 accepted species of *Thismia* Griff. (POWO 2019), all of which are small, diminutive herbs that lack chlorophyll. The genus is distributed across tropical and subtropical regions of Asia, Australasia and America, extending into some temperate regions (Yudina et al. 2021). Over a third of the known species of *Thismia* have been recorded to occur in Malaysia where the majority have very local distributions. These include 24 species in East Malaysia with six species in Sabah and 16 in Sarawak (Dančák et al. 2020a, b, c) and two species which occur in both states: *T. goodii* Kiew (Kiew 1999) and *T. brunneomitra* Hroneš, Kobrlová & Dančák (Hroneš et al. 2015). A further 15 species occur in Peninsular Malaysia (PM) (Jonker 1948; Siti-Munirah et al. 2021; Siti-Munirah and Dome 2021) including *T. clavigera* (Becc.) F.Muell. which occurs in both PM and Sarawak (Stone 1980; Dančák et al. 2020c).

Many species of *Thismia* have been seen or collected only once, from a single locality and their biology and ecology are poorly known. These elusive plants are visible only transiently, emerging to flower and set seed after heavy rain (Guo et al. 2019) and, like the related genus *Oxygyne* Schltr., are easily overlooked because they occur in deep shade, often under leaf litter (Thorogood 2019). Therefore, they can be difficult to relocate after their initial discovery. The extremely local distributions of mycoheterotrophs, like *Thismia*, may be restricted by host specificity for associated mycorrhizal fungi (Yamato et al. 2011; Gomes et al. 2017). The Malay Peninsula falls in the centre of diversity of *Thismia* and many new species have been described here in recent years, all of which are indeed very rare or local.

**Figure 1.**
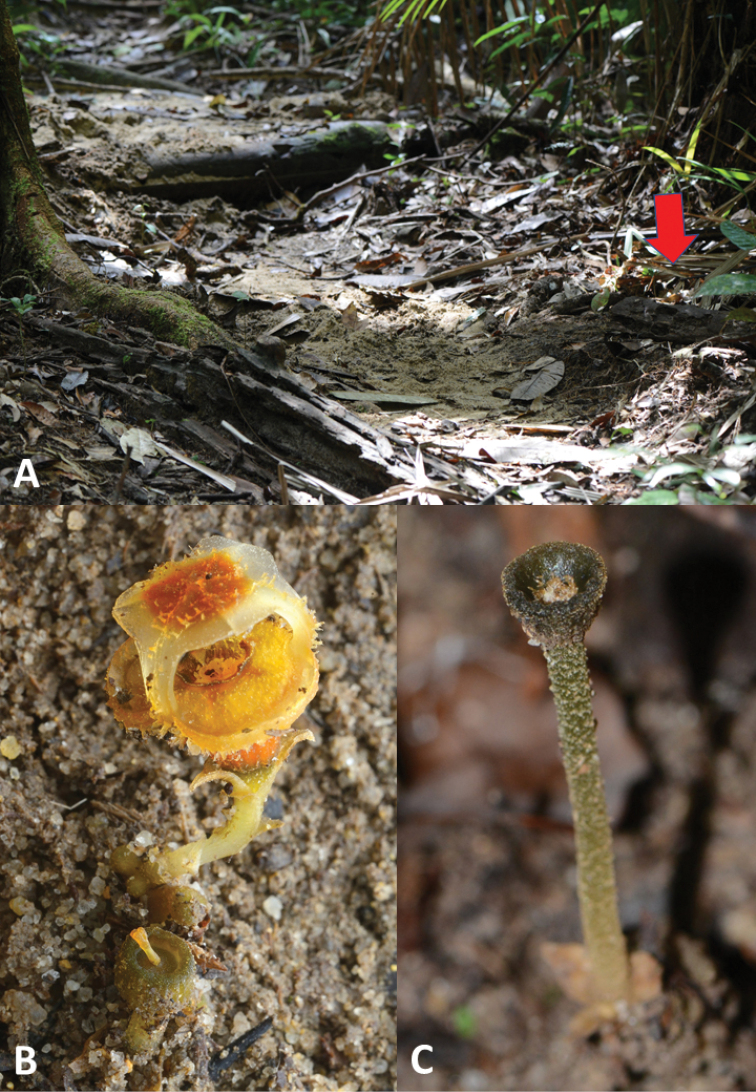
*Thismia sitimeriamiae***A** the habitat in Gunung Sarut, in the State of Terengganu (PM) **B** the habit of flowering specimen **C** a fruiting specimen (in situ).

An area of exceptional diversity of the genus *Thismia* is the Malaysian State of Terengganu, which contains a significant reservoir of primary rainforest, although this has diminished at a rapid and alarming rate in recent decades. To date, six species of *Thismia* have been reported in the State of Terengganu: *T. alba* Holttum & Jonker, *T. aseroe* Becc., *T. domei* Siti-Munirah, *T. javanica* J.J.Sm., *T. latiffiana* Siti-Munirah & Dome, *T. terengganuensis* Siti-Munirah (Jonker 1948; Siti Munirah and Dome 2019, 2021). This astonishing spate of species discoveries triggers the need for a reassessment of the genus in the Flora of Peninsular Malaysia and their conservation requirements. Here, we describe an additional new species which we name *Thismia sitimeriamiae* Siti-Munirah, Dome & Thorogood from the vicinity of Gunung Sarut, which is located in the Hulu Nerus Forest Reserve (FR) in the State of Terengganu. The plant was discovered in two sites in close proximity growing along the summit trail of the mountain (Fig. 1) by the second author in 2019. In February 2020, the authors revisited the locality during a botanical exploration of the Hulu Nerus FR led by the Forest Research Institute Malaysia (FRIM) Flora Team. Within the only known population, we found that one of the two sites had been destroyed by wild boar activity and the other had just a single fruiting specimen (Fig. 1). In December 2020, the second author revisited and observed just a single specimen after a thorough assessment of the area.

The distinctive structure, colour and morphology of this exceptionally rare and ephemeral plant make this species amongst the most eye-catching in the genus described from Peninsular Malaysia to date.

## Materials and methods

**Map 1.**
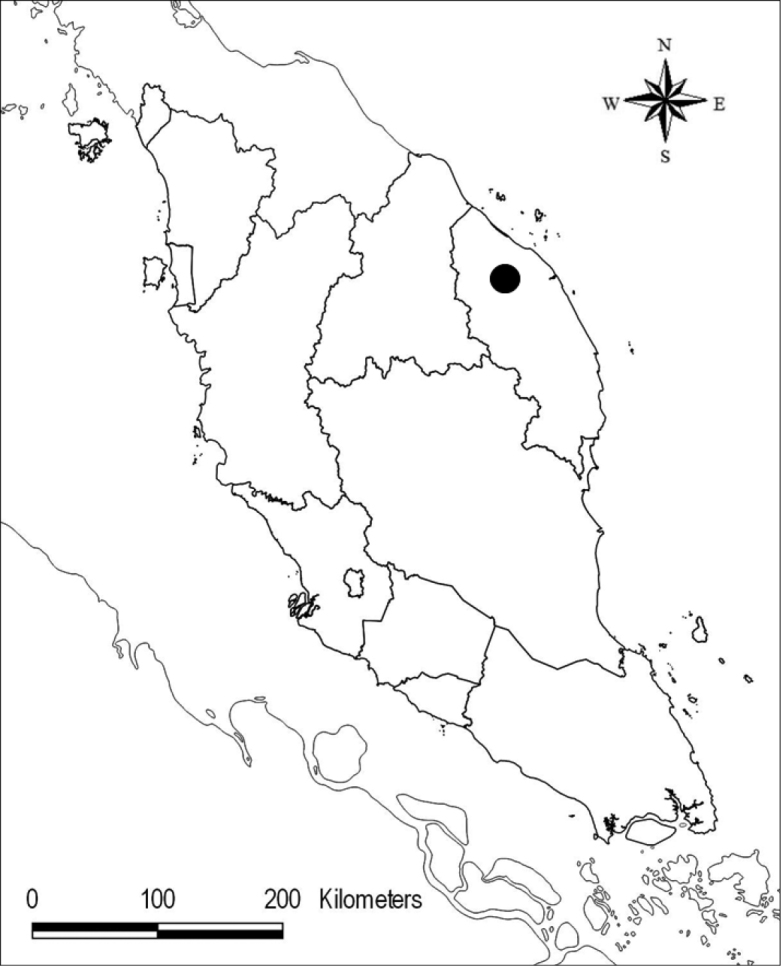
Hulu Nerus Forest Reserve (black circle), the type locality of *Thismia sitimeriamiae*.

Our assessment is based on material collected by the second author in December 2019 and 2020 from Hulu Nerus FR, Setiu District, Terengganu (Map 1). The specimens were preserved in 70% ethanol. In total, two specimens have been deposited in Kepong Herbarium (KEP) and have been examined for taxonomic treatment. Morphological characters and dimensions were examined using Olympus SZ61 and LEICA M125 stereomicroscopes and high-resolution macrophotography.

## Taxonomic account

### Thismia sitimeriamiae

Taxon classificationPlantaeDioscorealesBurmanniaceae

Siti-Munirah, Dome & Thorogood sp. nov.

A9F4F1C5-CFCB-5E0F-A805-EAB1492AB1CA

urn:lsid:ipni.org:names:77217986-1

**Figure 2.**
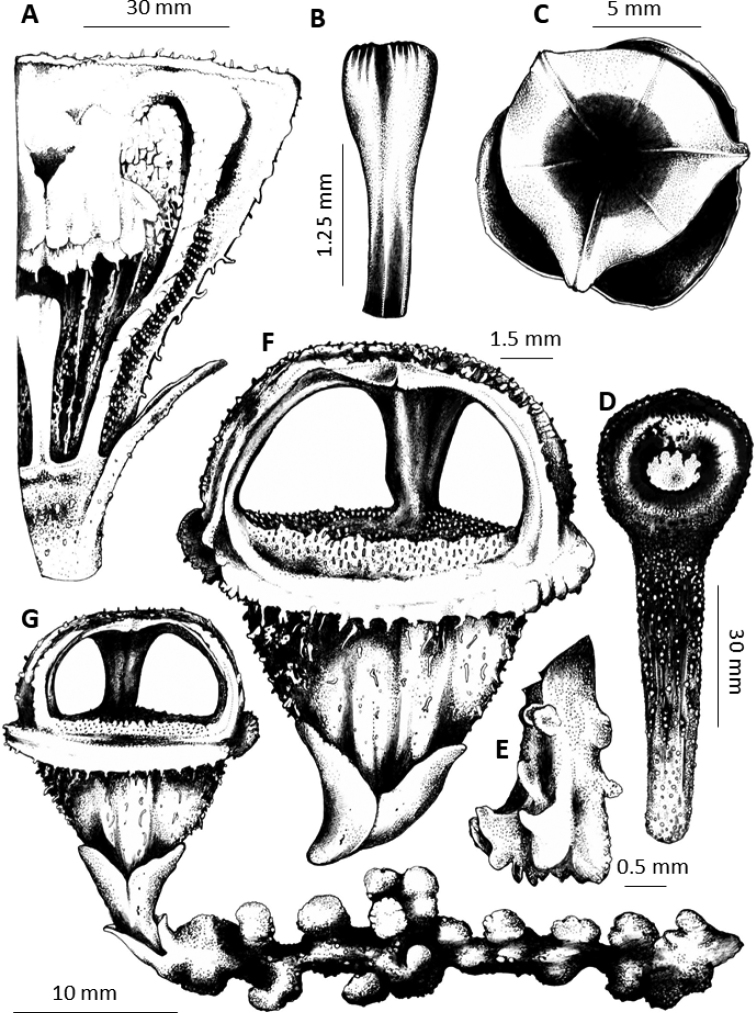
Illustration of *Thismia sitimeriamiae***A** cross section of perianth showing pendulous stamens (above) and slender pistil (beneath) **B** pistil **C** aerial view of flower showing mitre and portions of apical part of floral tube and outer tepals **D** fruit **E** stamen (showing outer side of connective) **F** flower, lateral view **G** habit, showing inflorescence (flower) and roots. All illustrated from *FRI 91118* (excluding **D**, in situ).

**Figure 3.**
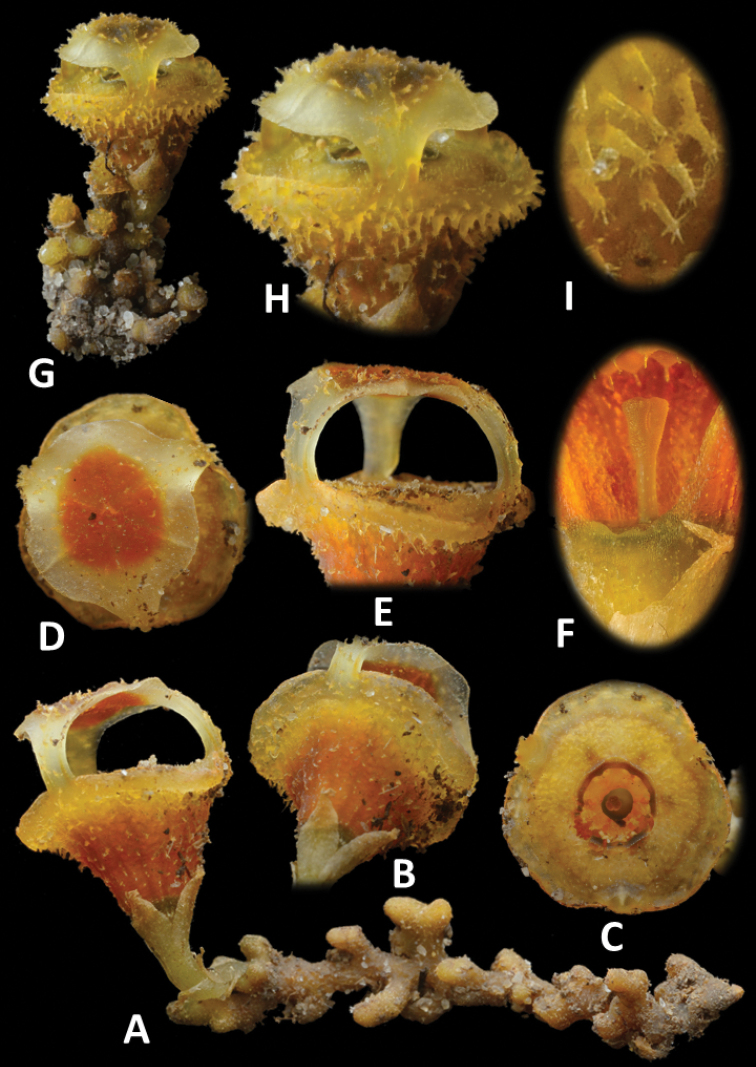
*Thismia sitimeriamiae***A** habit with roots **B** flower, view from below **C** aerial view of floral tube (mitre removed) **D** flower, aerial view **E** mitre, lateral view **F** ovary and pistil, lateral view **G** flower, lateral view **H** mitre, lateral view **I** stellate trichomes on the outer surface of floral tube. All from *FRI 91118* (**A–F**) and a plant photographed in situ (**G–I**). Images not to scale (see dimensions in description and Figure 2).

**Figure 4.**
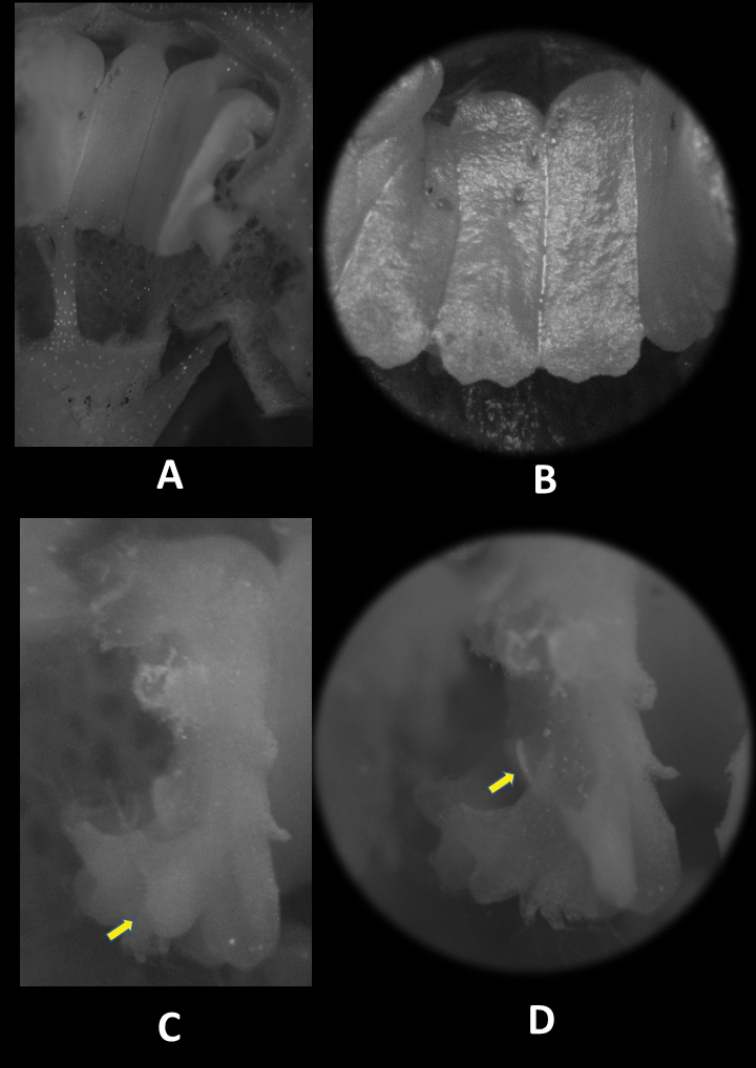
Flower structure of *Thismia sitimeriamiae***A** longitudinally dissected flower, showing inner side of stamens and pistil **B** stamens, (inner view) **C** lateral view of stamens showing lateral appendage (arrow) **D** lateral view of outer stamens in which interstaminal glands (arrow) are discernible. All photos from *FRI 91123*. For dimensions, see description and Figure 2.

Figures 1 , 2 , 3 , 4

#### Diagnosis.

*Thismia sitimeriamiae* has a unique combination of morphological traits, by which it differs from all its congeners including its orange colour, its surface characteristics and its floral architecture: the flower is strigose with pale orange simple and stellate trichomes covering the outer surface of the floral tube, mitre and ovary; the upper surface of the annulus is covered by very short trichomes; the outer tepals are reduced and inconspicuous and the inner tepals are curved upwards, connate, forming an umbrella/parasol-like mitre with a flattish apex.

#### Type.

Malaysia. Peninsular Malaysia: Terengganu, Setiu District, Hulu Nerus Forest Reserve, ca. 209 m alt., 23 December 2019, *Dome Nikong*, *FRI 91118* (holotype KEP!, spirit collection, No. barcode 280006).

#### Description.

Achlorophyllous herb, ca. 2.2 cm tall. ***Roots*** coralliform, creeping, light brown. ***Stem*** 8 mm tall, erect-ascending, unbranched, 2 mm thick, whitish. ***Leaves*** ca. 6, spirally arranged, triangular, scale-like, acute, entire, 3–4 mm long and 1 mm wide at base, pale whitish. ***Bracts*** 3, widely triangular to ovate, entire, acute to acuminate, ca. 5 mm long, 2 mm wide, pale greenish to brown. ***Pedicel*** 1 mm long, elongating after anthesis, greenish. ***Inflorescence* (flower)** solitary, actinomorphic, ca. 1.5 cm long; pale to dark orange; ***floral tube* (hypanthium)** conical in shape, ca. 1 cm in height, widest (1.2 cm) in the upper part; ***outer surface*** bright orange, sparsely covered with pale orange, simple trichomes (occasionally apically stellate); ***inner surface*** partially convex/reticulum; ***outer tepals*** 3, rather reduced and inconspicuous, divided, forming a narrow overhanging fringe around the mouth of the perianth tube; ***inner tepals*** well-developed, each pale orange, distally connate, forming a flattish, umbrella-like mitre ca. 3–4 mm tall, proximally flattened with revolute margins, arching over the floral tube; ***mitre*** circular in outline, ca. 8–10 mm wide, the upper surface bright orange, covered sparsely with orange trichomes, the lower surface glabrous, bright orange; ***annulus*** (apical part of the floral tube) pale orange, rounded, ca. 1.2 cm wide, the rim raised, connected with the inner tepals; upper surface covered with short trichomes; aperture, ca. 4–5 mm, with a blackish margin; ***stamens*** 6, orange, pendent from the annulus; ***filaments*** orange, curved downwards, ***connectives*** laterally connate, forming a tube, ca. 3 mm long, glabrous on the inner surface, the apex of each connective with two lobes, each pointed with transparent trichomes; ***outer side of connectives*** forming a lateral appendage, protruding towards the floral tube, horn-like on each side, shallowly dentate and sparsely hairy on the free margins; thecae pale, surrounded by tufts of hairs; ***interstaminal glands*** inserted along the line of fusion between the connectives; **ovary** inferior, obconical, flushed pale orange and greenish, covered by bracts; ***placentas*** 3, parietal; ***pistil*** slender, clavate, pale greenish-orange; ***style*** ca. 1.5 mm long, slender, erect, glabrous; ***stigma*** ca. 1 mm, papillose, 3-lobed, narrowly-rectangle, each without furrowed, orange; ***fruit*** a cup-shaped capsule, greenish, borne on an elongated pedicel up to 6 cm long; ***seeds*** brown.

#### Additional specimen examined

**(paratype).** Malaysia. Peninsular Malaysia: Terengganu, Setiu District, Hulu Nerus Forest Reserve, ca. 209 m a.s.l., 25 December 2020, *Dome Nikong FRI 91123* (KEP, spirit collection, barcode 28007).

#### Distribution.

Endemic to Terengganu, Peninsular Malaysia. Currently known only from the type locality (Map 1).

#### Ecology.

Lowland dipterocarp forest on moist soil in shade at an elevation of 209 m a.s.l. Flowering and fruiting from December to February.

#### Etymology.

*Thismia sitimeriamiae* is dedicated to Siti Meriam, the mother of the second author (Dome Nikong), in honour of her unparalleled support for the conservation activities pursued by Dome Nikong and her help in maintaining his plant collections.

#### Conservation status.

In accordance with the IUCN Red List Categories and Criteria (IUCN 2019), *Thismia sitimeriamiae* is assigned as Critically Endangered (CR), based on criterion B2 (ab (ii,iii)), because it is only known from the type locality, where fewer than five individuals have been observed (both in flower and in fruit), since survey work began.

## Discussion

### Taxonomy

*Thismia sitimeriamiae* is markedly distinct from all other species of *Thismia* reported to date. This species possesses unique morphological features including an outer floral surface covered with mixed simple and stellate trichomes, a connate mitre formed by the inner tepals with a flat elevated, umbrella-like portion and a pistil far more slender than those of other species described which normally possess a short and thick style. *Thismia sitimeriamiae* is morphologically most similar to *T. coronata* Hroneš, Dančák & Sochor (Dančák et al. 2020b) and *T. kelabitiana* Dančák, Hroneš & Sochor (Dančák et al. 2018) described from Sarawak, Malaysia. All three species possess yellow to orange flowers. However, *T. sitimeriamiae* differs in many traits, including its mitre structure and also the pistil shape which is more slender than those of *T. coronata* and *T. kelabitiana*; furthermore, the stigma of the latter two species is longitudinally furrowed, a pattern absent in *T. sitimeriamiae*. A detailed account of the morphological differences amongst the species is given in Table 1. The coralliform roots and presence of inner tepals connately fused to form a mitre indicate that *T. sitimeriamiae* belongs to Thismia sect. Sarcosiphon (Kumar et al. 2017). Based on recent molecular phylogenetic work carried out by Shepeleva et al. (2020), *T. sitimeriamiae* is likely to belong to a clade (denoted ‘clade 3’), characterised by species which possess coralliform roots and inner tepals fused into a mitre without foveae and includes the morphologically similar species *T. kelabitiana*. Further work should augment the molecular phylogenetic reconstruction of Shepeleva et al. (2020) to resolve the phylogenetic relationships of *T. sitimeriamiae* with related taxa.

**Table 1.** Morphological comparison of *Thismia sitimeriamiae*, *T. coronata* and *T. kelabitiana* (Dančák et al. 2018; Dančák et al. 2020b).

Character	*T. sitimeriamiae*	*T. coronata*	*T. kelabitiana*
**Height** (*cm*)	2.2	4.5–8	5–18
**Stem**			
*Length (cm)*	0.8	2.4–4.2	1.5–16
*Form*	simple	branched	branched
*Colour*	whitish	reddish-brown to orange	dark pinkish to reddish-brown (to almost grey or orange)
**Inflorescence (flower number)**	Solitary	1–6	1–3
**Leaves**			
*Number*	6	4–11	3–10
*Size*			
*Length (mm)*	3–4	2–5	4.5–5.5
*Width (mm)*	1	1–2	1.8–2
*Colour*	pale, whitish	light brown to reddish	light brown to pinkish
**Bracts**			
*Shape*	broadly triangular to ovate	lanceolate triangular	broadly triangular to ovate
*Margin*	entire	entire to irregularly dentate	entire to irregularly dentate, often deeply dissected
*Size*			
*Length (mm)*	5	4–8	6–8
*Width (mm)*	2	2–3	2.5–4
*Colour*	pale greenish to brown	reddish to brown	pinkish to brown
**Pedicel colour**	greenish	reddish-brown to orange	dark pinkish to reddish brown (to almost grey or orange)
**Flowers**			
*Size*			
*Length (cm)*	1.5	1.8–2.3	2.6–2.8
*Width (cm)*	1.2	0.7–1	1.2–1.3
*Colour*	pale to dark orange	dark yellow to orange	white to bright yellow
**Floral tube (hypanthium)**			
*Shape*	conical	funnel-shaped towards the base, apically urceolate	funnel-shaped towards the base, apically urceolate
*Colour*	outer surface more or less uniformly bright orange	dark yellow to orange, with 6 brownish-orange, prominent longitudinal ribs alternating with 6 brown-orange longitudinal stripes on outer surface	white to bright yellow at the top, with six brownish non-prominent longitudinal ribs and six yellow to brown longitudinal stripes on outer surface
*Surface texture*			
*Outer*	sparsely covered with pale orange, simple trichomes (occasionally apically stellate)	glabrous (trichomes absent)	glabrous (trichomes absent)
*Inner*	inner surface partly convex/reticulum	inner surface weakly reticulated, especially apically	inner surface reticulated
**Outer tepals**	3, rather reduced and inconspicuous, somewhat divided along the margin to form a narrow, inconspicuous fringe around the floral tube mouth	3, conspicuous, entire or slightly sinuate, often single-toothed, forming a fringe around the floral tube mouth	3, deeply divided into 8-10 acute lobes forming a conspicuous fringe around the floral tube mouth
**Inner tepals (and mitre)**	pale whitish-orange, distally connate, forming an elevated, more or less circular, umbrella-like mitre; lobes proximally flattened with revolute margins (not filiform), arched over the floral tube; upper surface covered sparsely with conspicuous orange trichomes	orange, distally connate, forming an elevated, more or less flat, triangular mitre; lobes proximally filiform, pillar-like, arched over the floral tube; trichomes absent	bright yellow to brownish-yellow, distally connate, forming a conspicuously elevated, more or less triangular mitre; lobes proximally filiform, pillar-like, arched over the floral tube; trichomes absent
**Annulus (apical part of floral tube)**	present (upper surface covered with short trichomes)	absent	absent
**Connectives**			
*Colour*	orange	white	white
*Length (mm)*	3	5–6	7
*Inner surface*	glabrous	with prominent longitudinal rib extending along inner side	with prominent longitudinal rib extending along inner side
*Apex of each connective*	2 lobes, each pointed with a transparent trichome	1 central lobe (extension of rib) and 2 smaller lobes, each lobe with a long transparent trichome	1 central lobe (extension of the rib) and 2 smaller lobes pointing somewhat centrifugally, each with a transparent trichome
*Outer surface*	lateral appendage protruding towards floral tube, horn-like on each side, shallowly dentate and sparsely hairy on free margins	lateral appendage box-shaped, protruding towards floral tube, not reaching apex of connective, shallowly dentate and hairy on apical margin, with tufts of hairs on lateral margins	lateral appendage box-shaped, protruding towards perianth tube, not reaching the apex of the connective, shallowly dentate and sparsely hairy on free margins
**Ovary**			
*Colour*	flushed pale orange and greenish	dark brown	dark reddish-brown
**Pistil**			
*Style*	slender	short	short
*Stigma*	lobes narrowly-rectangular, not furrowed; orange	lobes rectangular, longitudinally furrowed; dark brown	lobes rectangular, longitudinally furrowed; dark reddish-brown
**Capsule**			
*Colour*	greenish	pale brown to reddish at maturity	dark brown to blackish or reddish, maturing to become pinkish
**Pedicel**	elongated to 60 mm	10–25 mm	very short (dimensions not known)

We should note that our assessment of *T. sitimeriamiae* is based on a very limited sample due to the rarity of the plant and a paucity of material. Similar challenges were encountered for other species in this taxonomically difficult genus in PM, for example, *T. chrysops*, *T. fumida*, *T. racemosa* and *T. kelantanensis* (Jonker 1948; Siti-Munirah 2018). Indeed, the majority of descriptions of *Thismia* have been based on single collections due to a lack of accessibility of their habitats, the difficulty of finding them in flower or because of habitat loss and probable extinction. Whilst the type locality of *T. sitimeriamiae* is readily accessible, attempts to recollect the species in the site and in surrounding areas in Terengganu have so far proved fruitless.

### Reproductive ecology

The mating system and reproductive ecology of *T. sitimeriamiae* are unknown, as with most species of *Thismia*. Shepeleva et al. (2020) recommended a re-evaluation of the floral anatomy across the genus. The most detailed analysis of the floral architecture, anatomy and development of *Thismia* was carried out by Nuraliev et al. (2021). This work has identified an exceptional array of reproductive structures. The drivers of the exceptional floral diversity of *Thismia* are unknown, but possibly linked to their pollination biology. Mycoheterotrophs often produce inconspicuous flowers that are self-pollinated to maximise the seed set in areas where pollinators are scarce (Waterman and Bidartondo 2008; Guo et al. 2019). However, Mar and Saunders (2015) and Guo et al. (2019) identified outcrossing in species of *Thismia* and observed fungus gnats as the pollinators. We did not observe any floral visitors to *T. sitimeriamiae*; however, efforts to understand its reproductive biology would be impeded by its exceptional rarity.

### Conservation

We assess *T. sitimeriamiae* as Critically Endangered (CR B2 ab (ii,iii)) in accordance with the IUCN Red List Categories and Criteria because it is known only from the type locality, where just four individuals have been observed in total, including two flowering specimens in December 2019, one in fruit in February 2020 and a further specimen in flower in December 2020. The type locality is on a tourist trail within a forest reserve. Due to the sensitivity and human footfall of the habitat, the risk of disturbance is high. *T. sitimeriamiae* is probably unpredictable in appearance, like other species in the genus. Based on the authors’ long-term observations of the type locality and associated areas, the species is exceptionally rare or absent elsewhere. Furthermore, based on recent observations by the second author (June 2021), the only known location of the plant has been destroyed by wild boar activity, meaning that the species is now at considerable risk of extinction.

Mycoheterotrophic plants, such as *Thismia*, present an interesting challenge in plant conservation. Most are probably intractable to cultivation due to their very exacting ecologies and dependency on specific fungal partners. Botanic gardens could play a part in the conservation of *Thismia* as they have for other intractable plants. For example, botanic gardens have played a role in the conservation of the mycoheterotrophic orchid *Rhizanthella gardneri* in Australia through survey work and successful transplant and propagation (Swarts and Dixon 2009; Thorogood et al. 2019). In the Philippines, conservation of parasitic *Rafflesia* has involved increasing the availability of host plants in fragmented habitats with rooted *Tetrastigma* cuttings (Pelser et al. 2017). Creative approaches may, therefore, be required for conserving rare and poorly understood species with complex ecological interdependencies. Given the rarity and inaccessibility of the vast majority of species of *Thismia* (many of which have been found only once), *in situ* conservation seems to be the only realistic approach. Survey work coupled with taxonomic assessment will provide information for such an approach.

### Concluding remarks

Since the most comprehensive taxonomic revision of the genus *Thismia* in the 1930s and 40s (Jonker 1938, 1948), the number of species described in this genus has proliferated. Recent species discoveries and investigations of the floral anatomy and evolution of the genus have brought an astonishing diversity of floral structure to our attention (Shepeleva et al. 2020; Nuraliev et al. 2021). The extraordinary morphology and extreme scarcity of *Thismia sitimeriamiae* raise interesting questions about the ecology, evolution and conservation of this and other species in this exceptional genus. Further survey work and taxonomic assessment will provide information for the setting of conservation priorities for these mysterious plants, many of which have been seen only once and some may never be seen again.

## Supplementary Material

XML Treatment for Thismia sitimeriamiae
